# Cell surface surprise: NPM1 is an immune therapy target for acute myeloid leukemia

**DOI:** 10.1002/hem3.70193

**Published:** 2025-08-07

**Authors:** Hansen J. Kosasih, Charles E. de Bock

**Affiliations:** ^1^ Lowy Cancer Research Centre, Children's Cancer Institute UNSW Sydney Kensington New South Wales Australia; ^2^ School of Clinical Medicine UNSW Sydney Kensington New South Wales Australia

Our improved understanding of genetic mutations and subtypes in acute myeloid leukemia (AML) has improved risk‐stratification and shaped the development of targeted small‐molecule inhibitors now used clinically. This includes venetoclax (BCL2 inhibitor) combined with decitabine (DNMT3A inhibitor) for older AML patients, gilteritinib (FLT3 inhibitor) for newly diagnosed *FLT3*‐mutated AML, and enasidenib (IDH2 inhibitor) for relapsed or refractory *IDH2*‐mutated AML.^1^ However, immunotherapy development for AML has been more challenging and has not replicated the remarkable success achieved in B‐cell acute lymphoblastic leukemia (B‐ALL).

This disparity in immunotherapy success stems in part from the fundamental difference in target selection. In B‐ALL, immunotherapies target common cell lineage surface antigens such as CD19, CD20, or CD22. Although this means that both normal B cells and malignant cells are eliminated, the resulting B‐cell aplasia is not immediately life‐threatening and can be clinically managed by regular intravenous immunoglobulin replacement therapy. This is in contrast with AML, where targeting common myeloid lineage antigens results in profound on‐target off‐tumor toxicity. This is exemplified by gemtuzumab ozogamicin, currently the only European Medicines Agency and US Food and Drug Administration (FDA)‐approved immunotherapy, which causes significant on‐target toxicity because its target, CD33, is expressed not only on AML blasts and leukemic stem cells but also on healthy hematopoietic stem/progenitor cells (HSPCs), granulocytes, and monocytes.

As the field pursues new immunotherapies for AML, a recent review highlighted a total of 34 different cell surface antigens targeted with chimeric antigen receptor (CAR)‐based strategies, with CD33, CD123, and CLEC12A making up 58% of the clinical CAR reports.^2^ Unfortunately, similar to CD33, CD123 is expressed on HSPCs, monocytes, dendritic cells, and B cells. CLEC12A (also known as CLL‐1) represents a more promising target because its absence on normal stem cells, while being expressed heterogeneously on AML cells. Nevertheless, CAR therapies targeting CD33, CD123, or CLEC12A have not yielded durable responses in most AML patients, often causing significant toxicities and being further limited by antigen escape as a key resistance mechanism.^2^ Therefore, identifying cell surface targets on AML cells that can be therapeutically targeted while minimizing on‐target, off‐tumor toxicity on healthy tissues remains a critical research priority.

Given the limitations of targeting common myeloid antigens, there is a growing interest in personalizing AML immunotherapies by exploiting patient‐specific mutations. One promising strategy is targeting unique mutations found in intracellular proteins that are subsequently presented as neoantigens on the cell surface as part of the major histocompatibility complex. A broad investigation of 14 recurrent driver mutations identified IDH2^R140Q^ as the immunogenic neoantigen, with IDH2^R140Q^‐specific neo‐T cells showing significant antitumor effects in preclinical mouse models.^3^ Another common recurrent mutation in AML is in the RNA‐binding protein NPM1, resulting in a frame shift and addition of 11 amino acids to the C‐terminus that retains it in the cytoplasm (NPM1c). From a prognostic perspective, NPM1 mutations alone are associated with a more favorable prognosis, but this shifts to adverse risk when accompanied by additional mutations, particularly FLT3‐ITD and DNMT3A^R882^.^4^, ^5^ An early study found that a mutant NPM1c peptide covering the alternate C‐terminus was expressed as a neoantigen on the surface of AML cells and could be efficiently targeted through T‐cell receptor gene transfer into T cells, resulting in a strong antitumor response.^6^ Given the frequent and recurrent mutations in splicing factors and general dysregulation of RNA‐binding proteins, including NPM1 in AML, additional neoantigens likely exist that could be targeted by a similar approach.^7^ However, although this neoantigen approach offers the promise of a bona‐fide personalized therapy with minimal off‐target toxicity, it remains largely experimental. Therefore, more conventional CAR‐T or antibody–drug conjugate strategies continue to represent a more viable therapeutic option for rapid translation into the clinic.

In a new study seeking to address the limitations of current immunotherapies, George et al.,^8^ took a novel approach and surveyed the surface proteome of cells with a focus on RNA‐binding proteins, which had previously found to be on the cell surface. Surprisingly, they identified cell surface expression of NPM1 across seven different datasets. Using molecular biology techniques and super‐resolution imaging, the authors confirmed the presence of NPM1 at the cell surface of AML human cell lines and murine models of AML, in both NPM1 and NMP1c AML.

To therapeutically exploit this cell surface expression, the authors engineered a new anti‐NPM1 antibody with a mouse Ig2a backbone to maximize antibody‐dependent cellular cytotoxicity (ADCC), simply called “mAb2” (Figure 1). This antibody specifically bound to cell surface NPM1 (csNPM1), and CRISPR/Cas9‐mediated knockout of NPM1 on AML cell line abolished mAb2 binding, confirming target specificity. Using this antibody as a staining tool, the authors found that between 21% and 54% of blast cells displayed detectable csNPM1 across 31 primary patient AML samples.

**Figure 1:**
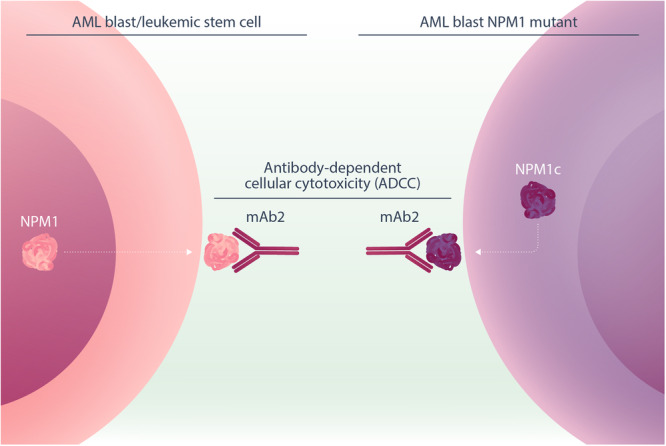
**Schematic illustrating the cell surface localization of NPM1 and mutant NMP1c on blast cells or leukemic stem cells.** Binding of mAb2 to cell surface NPM1 or NPM1c then results in antibody‐dependent cell cytotoxicity (ADCC) within preclinical leukemia models in vivo. (AML, acute myeloid leukemia).

Importantly, mAb2 demonstrated significant therapeutic efficacy in vivo across two different primary murine AML models (NPM1^cA/+^; Flt3^ITD/+^ AML and MLL‐AF9/Flt3^ITD/+^AML) and a human MLL‐rearranged patient‐derived xenograft model. Treatment with mAb2 significantly reduced leukemia burden and extended overall survival. Importantly, from a clinical perspective, mAb2 treatment also significantly reduced the leukemia stem cell compartment.

To assess potential on‐target off‐tumor toxicity from targeting csNPM1, the authors examined its expression on normal hematopoietic cells. They found that CD33+ myeloid cells had a 10‐fold lower expression, and CD34+ HSPCs showed a 100‐fold lower expression compared to AML blasts. More importantly, when the mAb2 antibody, which also recognizes and cross‐reacts with murine cell surface NPM1, was injected into C57BL/6 mice, no change in blood counts was observed. This suggests minimal on‐target off‐tumor side effects, albeit more work still needs to be done for non‐hematopoietic tissues.

This study provides compelling evidence for targeting NPM1 as a new therapeutic target for AML immunotherapy. This surprising finding of cell surface NPM1 and the development of an effective targeting antibody offers several key advantages including (i) this therapy being AML genotype agnostic; (ii) targets the leukemic stem cell fraction; and (iii) has a potentially favorable safety profile with minimal toxicity to healthy normal hematopoietic cells. Furthermore, given that mutant NMP1c is an oncogenic driver and cell dependency, refining the targeting antibody to specifically target the mutant NPM1c would also prevent antigen escape as a resistance mechanism. Beyond the immediate findings of this paper, the authors also make a strong argument to not immediately discount noncanonical cell surface proteomic results as just “noise” and exemplify how “believing the data” can uncover a novel therapeutic target for AML.

## AUTHOR CONTRIBUTIONS

**Hansen J. Kosasih**: Conceptualization; writing—original draft; writing—review and editing. **Charles E. de Bock:** Conceptualization, writing—original draft; writing—review and editing.

## CONFLICT OF INTEREST STATEMENT

The authors declare no conflicts of interest.

## FUNDING

No funding was received for this publication.

## Data Availability

Data sharing is not applicable to this article as no datasets were generated or analyzed during the current study.
